# The planthopper genus *Spartidelphax*, a new segregate of Nearctic *Delphacodes* (Hemiptera, Delphacidae)

**DOI:** 10.3897/zookeys.453.8369

**Published:** 2014-11-10

**Authors:** Charles R. Bartlett, Mick D. Webb

**Affiliations:** 1 Department of Entomology and Wildlife Ecology, University of Delaware, 250 Townsend Hall, 531 S College Ave., Newark, DE 19716-2130; 2 Department of Entomology, The Natural History Museum, South Kensington, London SW7 5BD, UK

**Keywords:** New genus, Delphacidae, planthopper, Fulgoroidea, Auchenorrhyncha, Hemiptera, Poaceae, *Spartina*, *Delphacodes*

## Abstract

The new genus *Spartidelphax* is described to house three species removed from the polyphyletic genus *Delphacodes*. The members of *Spartidelphax* are coastal species native to eastern North America, and probably feed exclusively on cordgrass (Poaceae, *Spartina* Schreb.). The taxonomy and nomenclature of the included species (viz. *Spartidelphax detectus*, *Spartidelphax luteivittus*, and *Spartidelphax penedetectus*) are reviewed. *Spartidelphax luteivittus* is a *nomen dubium*, whose type material is inadequate to provide diagnostic features contrasting with *Spartidelphax detectus* and *Spartidelphax penedetectus*. Diagnoses and a key are provided for the remaining *Spartidelphax*.

## Introduction

*Delphacodes* Fieber, 1866, is a polyphyletic genus (e.g., Urban et al. 2010) with approximately 158 nominative species worldwide at this time (Bourgoin 2014, Bartlett 2014). *Delphacodes* *sensu stricto* is composed of 10 species from the western Palearctic (Asche and Remane 1983). The three North American “*Delphacodes*” species, *Delphax luteivitta* (Walker), *Delphacodes detecta* (Van Duzee) and *Delphacodes penedetecta* Beamer, are morphologically similar (e.g., Beamer 1950), and the latter two have been phylogenetically placed within the advanced Delphacini as basal to the clade of *Prokelisia* + *Neomegamelanus* + *Tumidagena* (collectively called the *Spartina*-clade) by Urban et al. (2010). This clade is predominately coastal and cordgrass-feeding (*Spartina* Schreb., Poaceae). The coastal marsh planthopper fauna has been extensively studied in a variety of ecological-evolutionary contexts (e.g., Davis and Gray 1966, Denno 1976, 1977, 1978, 1980, Raupp and Denno 1979, Denno et al. 1980, Rey 1981, Olmstead et al. 1997, Ferrenberg and Denno 2003). *Delphacodes detecta* has been reported along the Atlantic coast from Canada to Florida and the Gulf coast to Texas plus the Caribbean (Beamer 1950, Bartlett et al. 2014), where it can be abundant. Denno (1978) reported 23,868 collected over a year from *Spartina patens* (Aiton) Muhl. in New Jersey. *Delphacodes penedetecta* has been reported from the Gulf Coast (AL, FL, LA, MS, TX) and New Jersey. This species probably occurs along much of the east coast, but is evidently uncommon because of competition with the abundant *Prokelisia dolus* Wilson, 1982 on *Spartina alterniflora* Loisel. (Ferrenberg and Denno 2003).

Although the species described as *Delphax luteivitta* Walker, 1851, appears to be related to *Delphacodes detecta* and *Delphacodes penedetecta* its identity cannot be reliably ascertained due to the poor condition of its type (see below). It was described from a single male specimen from ‘United States’ (“presented by E. Doubleday”) as being straw-colored, with a produced head and dark front bordered by pale straw (Walker 1851: 354). It was subsequently transferred to *Dicranotropis* (with uncertainty) by Van Duzee (1916). Metcalf (1923: 148) excluded it from his treatment of eastern planthoppers because “the male genitalia have not been described”. Subsequently, Muir and Giffard (1924: 12) provided a brief description of the genitalia and transferred it to *Stenocranus*. Beamer (1946: 1) placed the species into *incertae sedis*, commenting “…judging from descriptions and drawings of the type in the British Museum by W. E. China, [it] does not belong in *Stenocranus*.” Bartlett (2010: 472) reported that the type specimen labels consist only of “the registration number on a circular white label clockwise from left “5 41 17 229.1”, indicating entry 229 of the 17th May 1841”, and that the Doubleday specimens were from St. Johns Bluff, Florida (Duval County, near Jacksonville; based on communications from M. Webb, British Museum Natural History, and K.G.A. Hamilton, Canadian National Museum). Bartlett (2010: 473) transferred *luteivitta* to *Delphacodes*, and suggested that it may be conspecific with a subsequently described species of that genus, although “further investigation will be needed to firmly establish the synonymy and explore nomenclatural implications”.

Here we investigate the taxonomy and nomenclature of *Delphacodes detecta*, *Delphacodes penedetecta* and *Delphax luteivitta*. Each species is photographed and illustrated, and a diagnosis and key are provided. A new genus is described to partition them from the western Palearctic *Delphacodes* *sensu stricto*.

## Materials and methods

Specimens were examined from the following collections:

AMNH American Museum of Natural History, New York, NY.

BMNH The Natural History Museum, London, U.K.

DENH University of New Hampshire, Department of Entomology, Durham, NH.

ISUI Iowa State University Insect Collection, Department of Entomology, Ames, IA.

LSUC Louisiana State University Arthropod Museum, Baton Rouge, LA.

NCSU North Carolina State University, Department of Entomology, Raleigh, NC.

SEMC University of Kansas Biodiversity Institute, Snow Entomological Museum Collection, Lawrence, KS.

UDCC University of Delaware Insect Research Collection, Newark, DE.

URIC University of Rhode Island Insect Collection, Department of Plant Sciences and Entomology, Kingston, RI.

USNM National Museum of Natural History (United States National Museum), Washington D.C.

Diagnoses are provided for each species emphasizing putatively distinguishing features (full descriptions of *detecta* and *penedetecta* were provided by Beamer 1950). For the diagnoses, topotypic paratype males of *Delphacodes penedetecta* (Cedar Keys, FL) were dissected and illustrated and compared to available specimens of *Delphacodes detecta*. The male lectotype (designated by Oman 1947: 211) of *Delphacodes detecta* could not be located for this study, although the female paralectotype was found (Figure 5; at ISUI) and included. *Delphacodes luteivitta* (Walker) is recorded only from the holotype in the British Museum (Natural History). For primary types, labels were quoted verbatim using “/” to indicate a line break and “//” to indicate a new label and with supplemental information given in brackets. For other material examined, label data are arranged into a consistent sequence, beginning with country, state or province, specific locality, collection date, and collector, with number, gender (as ‘m’ for males, ‘f’ females) and specimen depository given in parentheses. Specimens examined were provided 2D barcode labels and data were captured for online presentation (visualized at discoverlife.org and iDigBio.org) using “Arthropod Easy Data Capture” (Schuh et al. 2010, Schuh 2012, Arthropod Easy Capture 2013).

Photographs and measurements of *Delphacodes detecta* and *Delphacodes penedetecta* were taken using a digital imagery system consisting of a Nikon SMZ1500 microscope, Nikon Digital Sight DS-U1 camera and NIS Elements Imaging software (version 3.0). Line art was digitally traced from photographs. All measurements are in millimeters (mm).

The holotype of *Delphax luteivitta* (as the BMNH) was examined and photographed (by MDW) to assess features of this specimen in comparison to *Delphacodes detecta* and *Delphacodes penedetecta*. Photographs were taken using a Leica M125 Stereomicroscope, Canon Digital EOS 550D camera with EOS Utility and Helicon Focus software.

Morphological terminology follows Asche (1985, 1990) and subsequent authors (e.g., Bartlett and Deitz 2000, Gonzon and Bartlett 2008, Bartlett and Hamilton 2011, Bartlett et al. 2014). Plant names are from USDA PLANTS database (USDA, NRCS 2014).

## Results

### Systematics

#### Spartidelphax gen. n.

Taxon classificationAnimaliaHemipteraDelphacidae

http://zoobank.org/FC460372-49D0-41E7-A9B1-449274706188

##### Type species.

*Delphacodes penedetecta* Beamer, 1950.

##### Diagnosis.

Body robust, stramineous with dark markings on intracarinal region of face (anterior to the Y-shaped carina of vertex), including areolet, genae, and usually also lateral portions of abdominal terga. Body not compressed (unlike *Prokelisia*). Head, including compound eyes, slightly larger than pronotum, vertex in dorsal view weakly projecting between eyes. Carinae of head strong and conspicuous, except median carina of vertex; median carina of frons forked on fastigium near dorsal margin of compound eye. Frons with lateral margins subparallel, narrowed between eyes. Lateral carinae of pronotum diverging, not reaching posterior margin; median carina reaching hind margin at shallow notch. Lateral carinae of mesonotum diverging, reaching posterior margin, median carina becoming obsolete in scutellum. Forewings of brachypter clear, subtruncate, leaving several tergites exposed. Apex of hind tibiae bearing 7 (3+4) spines, with 5 (2+3) on basitarsus and 4 on second tarsomere. Calcar with 18–31 teeth (x = 24.0, n=26).

Male terminalia with pygofer rather quadrate in lateral view, dorsocaudal margin of pygofer weakly projecting. Opening of pygofer broad, wider than long, with lateral margins of opening carinae, ventral margin smoothly rounded. Diaphragm strong and conspicuous, dorsal margin broadly U-shaped, bearing median, bilobed armature subtending the aedeagus, much wider than tall. Parameres exerted through broad opening in diaphragm; parameres strongly flattened, sides subparallel, strongly diverging, basal and apical angles weakly developed. Aedeagus widest in basal third, then abruptly narrowed with distal 2/3 strongly downcurved; suspensorium U-shaped, weakly apparent. Segment 10 broad, bearing strongly developed pair of weakly sinuate processes on caudal margins near lateral margins. Segment 11 about 2/3 height of segment 10.

Macropters darker than brachypters, with abdomen and lateral portion of mesonotum more strongly embrowned. Macropterous wings are clear (no dark marking at apex of clavus), exceeding length of abdomen nearly by length of abdomen.

##### Remarks.

*Spartidelphax penedetectus* was chosen as the type species since the holotype of *Delphax luteivitta* is in unsatisfactory condition and the lectotype of *Liburnia detecta* could not be located (although putatively at the USNM). The holotype of *Delphacodes penedetecta* Beamer, 1950, is at SEMC.

*Spartidelphax* is phylogenetically placed at the base of a strongly supported clade with the genera *Prokelisia* Osborn, *Neomegamelanus* McDermott, and *Tumidagena* McDermott based on the phylogenetic investigation of Delphacidae using DNA nucleotide sequence data from four genetic loci (18S rDNA, 28S rDNA, wingless and cytochrome oxidase I) and 132 coded morphological characters by Urban et al. (2010). These three genera and *Spartidelphax* are associated with *Spartina* Schreb. (Poaceae, cordgrass), and are abundant in salt marshes in eastern North America. *Prokelisia*, *Neomegamelanus*, and *Tumidagena* are more slender forms with their body weakly to strongly compressed, and their vertex more strongly projecting. Members of *Prokelisia* are most similar, including having the carinae on their frons bordered by dark (except *Prokelisia crocea*), but they are more slender, usually with the frons broadest ventrally, parameres either distally converging or slender and diverging, and the aedeagus is usually upturned. Superficially more similar to *Spartidelphax* are species now placed in *Muirodelphax* Wagner, but North American species in this genus lack processes on segment 10. Also similar are *Toya* Distant, *Metadelphax* Wagner, and *Syndelphax* Fennah, but the dorsocaudal angles of the male pygofer of these genera are greatly expanded (Gonzon and Bartlett 2008).

In the “Key to genera of Delphacidae North of Mexico” of Bartlett et al. (2014), *Spartidelphax* keys to couplet 75, where *Spartidelphax* can be inserted in place of the entry for *Delphacodes detecta* and *Delphacodes penedetecta*.

##### Etymology.

The generic name is an arbitrary combination of letters formed by combining a truncation of *Spartina* (the host grass genus) with -*delphax*, a common termination used in delphacids. The name is to be treated as masculine (*Delphax* was affirmed as masculine by ICZN 1961).

##### Key to species of *Spartidelphax* (males)

**Table:** 

1	Aedeagus with ventral teeth or fine serrulations (Fig. 4D); vertex nearly 1.5× longer than wide; body length (brachypterous male) 2.18–2.57 mm	***Spartidelphax penedetectus***
–	Aedeagus with long rows of lateral teeth extending beyond distal third of aedeagus (Fig. 4B); vertex usually 1.3× longer than wide; body length (brachypterous male) 1.89–2.43 mm	***Spartidelphax detectus***

#### Spartidelphax penedetectus

Taxon classificationAnimaliaHemipteraDelphacidae

(Beamer, 1950) comb. n.

**Figure 1.**
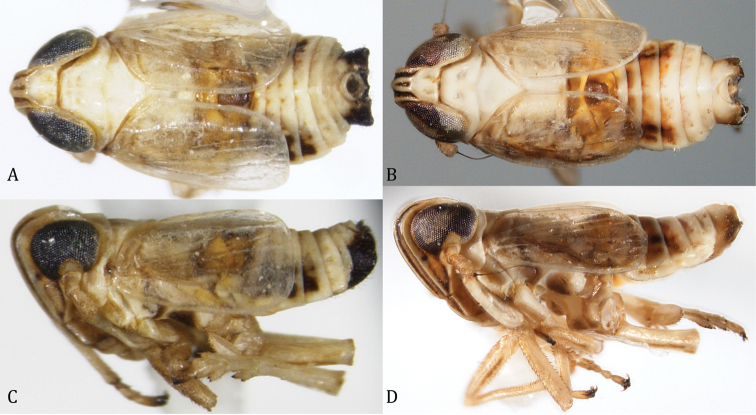
Dorsal and lateral views of *Spartidelphax detectus* (New Castle Co., DE) and *Spartidelphax penedetectus* (Franklin Co., FL). **A** Dorsal view of *Spartidelphax detectus* **B** same *Spartidelphax penedetectus*; **C** lateral view of *Spartidelphax detectus* **D** same, *Spartidelphax penedetectus*.

**Figure 2.**
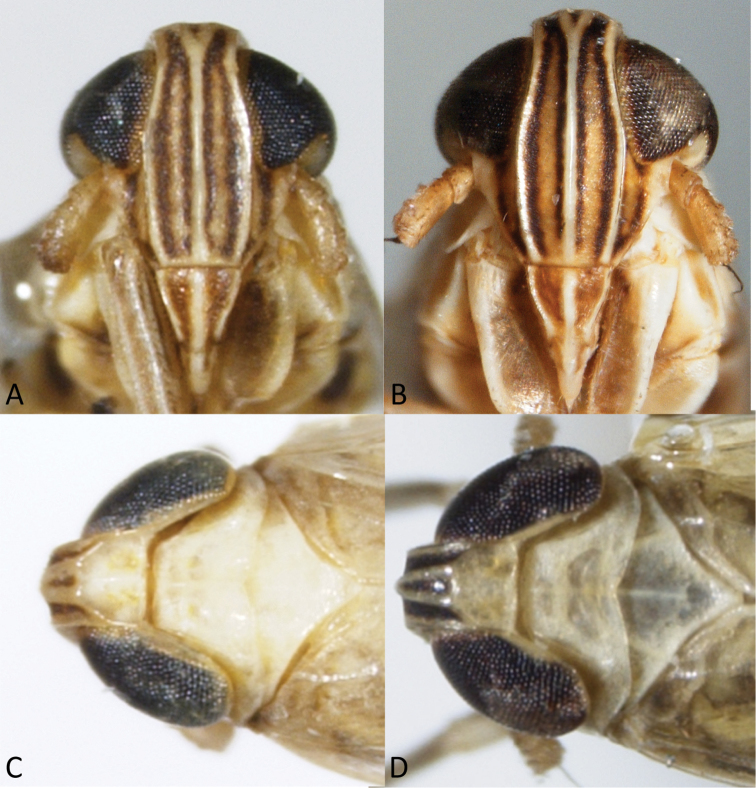
Heads of *Spartidelphax detectus* (New Castle Co., DE) and *Spartidelphax penedetectus* (Franklin Co., FL). **A** Frontal view of *Spartidelphax detectus* **B** same *Spartidelphax penedetectus* **C** dorsal view of head and anterior thorax of *Spartidelphax detectus* **D** same, *Spartidelphax penedetectus*.

**Figure 3.**
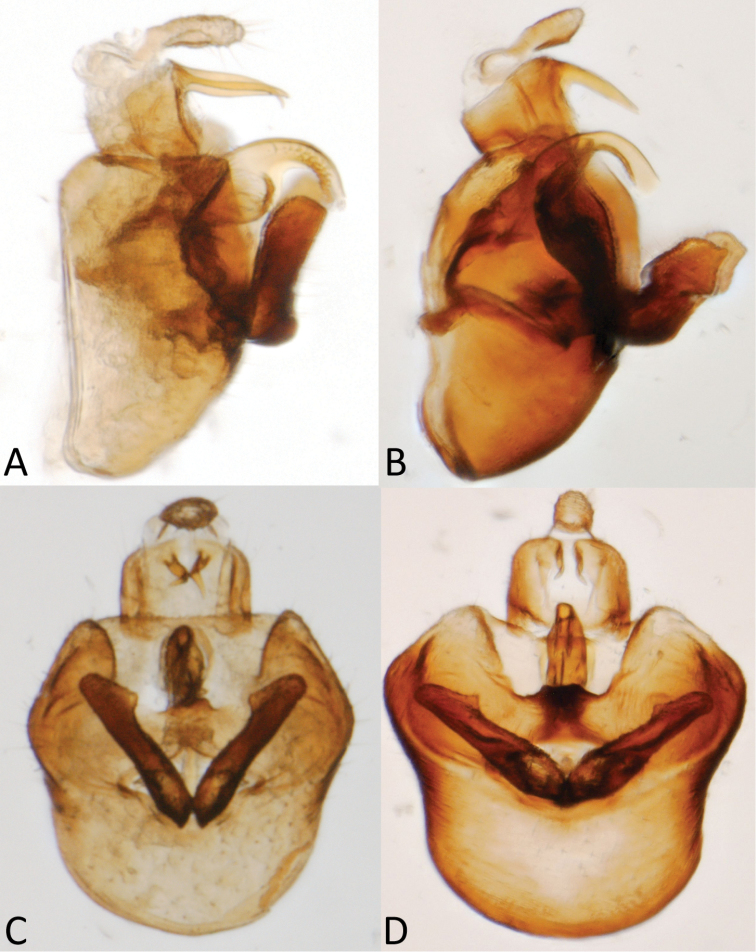
Male terminalia of *Spartidelphax detectus* (Kent Co., DE) and *Spartidelphax penedetectus* (topotypic paratype, Cedar Keys, FL). **A** Lateral view of *Spartidelphax detectus* **B** same *Spartidelphax penedetectus* **C** caudal view of head and anterior thorax of *Spartidelphax detectus* **D** same, *Spartidelphax penedetectus*.

**Figure 4.**
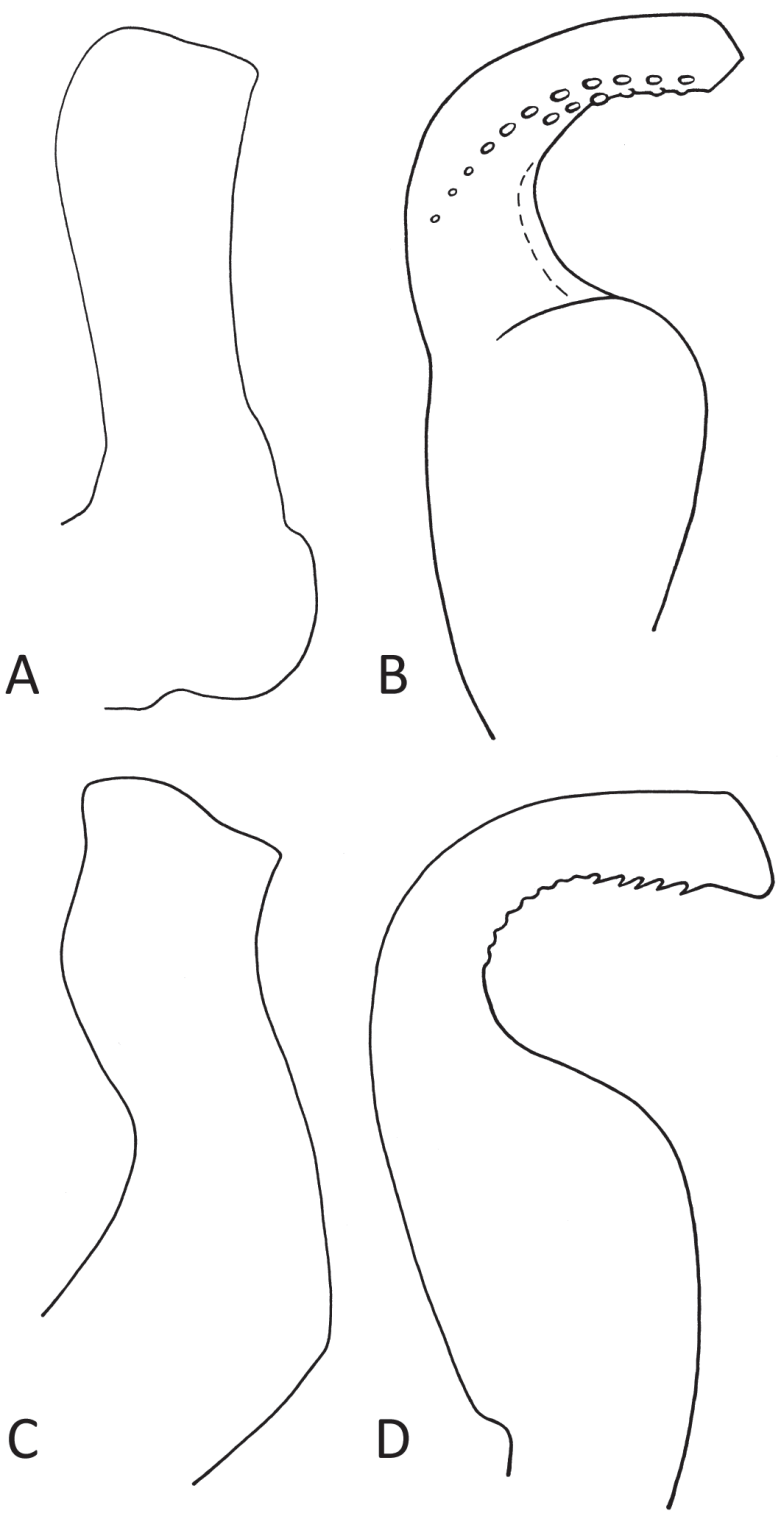
Line art of left paramere (widest view) and aedeagus (rotated 90° clockwise, apex up) of *Spartidelphax detectus* (Sanford, FL) and *Spartidelphax penedetectus* (paratype, Cedar Keys, FL). **A** Paramere of *Spartidelphax detectus* **B** aedeagus of *Spartidelphax detectus* **C** paramere of *Spartidelphax penedetectus* **D** aedeagus of *Spartidelphax penedetecus*.

Figures 1B, D ; 2B, D ; 3B, D ; 4C, D

Delphacodes penedetecta Beamer, 1950: 70.

##### Type locality.

Florida, Levy County, Cedar Keys.

##### Diagnosis.

Slightly larger than *Spartidelphax detectus*, with vertex longer than wide (l:w 1.34–1.48), aedeagus with a pair of rows of fine ventral serrulations in distal third; base less abruptly narrowed than in *Spartidelphax detectus*. Parameres in widest view subtly more narrowed on outer angle than *Spartidelphax detectus*, outer angle slightly curled.

*Dimensions.* Male brachypter: body length 2.33 mm (2.18–2.57, n=6), vertex l:w (1.48, n=9); male macropter: body Length 3.79 (including wings, 3.62–3.96, n=6), vertex l:w (1.44, n=6). Female brachypter: body length 3.06 (2.87–3.27, n=6), vertex l:w (1.34, n=6); female macropter: body length 4.07 mm (3.62–4.45, n=4), vertex l:w (1.39, n=5). Count of calcar teeth 25 (21–31, n=10).

##### Reported hosts.

*Spartina alterniflora* Loisel. (smooth cordgrass) (Wilson et al. 1994, Ferrenberg and Denno 2003).

##### Distribution.

USA: FL, LA, NC, TX; also reported AL, MS, NJ (Ferrenberg and Denno 2003, Bartlett et al. 2014).

##### Type material examined.

**Paratypes:** “Cedar Keys. Fla. / 3-8-1947 / R. H. Beamer // ♂[yellow paper] // Paratype / Delphacodes / penedetecta / R. H. Beamer” (2m, SEMC).

##### Other material examined.

**USA: Florida: *Franklin Co.*:** Ochlockonee Bridge, Highway 98 near Panacea, 29.96884°N, 84.38366°W, 27 Jul 2000, C. R. Bartlett (10m, 6f; UDCC). **Louisiana: *Cameron Par.*:** Cameron Parish, 03 Apr 1974, no collector provided (1m, 1f; LSUC); 15 Apr 1974, no collector provided (2m; LSUC); Holly Beach, 27 May 1983, E. G. Riley (3f; LSUC); same, 20 Apr 1984, D. A. Rider (1m; LSUC). **North Carolina: *Carteret Co.*:** near Atlantic, drum inlet, 19 Aug 1975, N. Newton (1m; UDCC).

#### Spartidelphax detectus

Taxon classificationAnimaliaHemipteraDelphacidae

(Van Duzee, 1897) comb. n.

**Figure 5.**
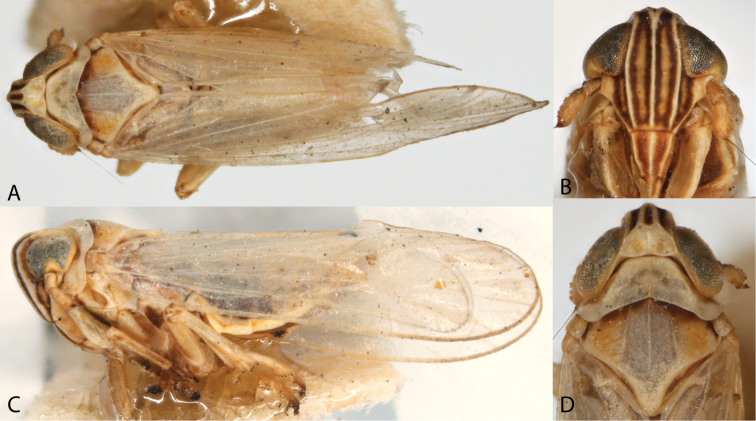
Female paralectotype of *Liburnia detecta* Van Duzee, 1897 (New York, NY). **A** dorsal habitus **B** front **C** lateral habitus **D** dorsal view head and anterior thorax.

Figures 1A, C ; 2A, C ; 3A, C ; 4A, B , 5

Liburnia detecta Van Duzee, 1897: 248.
Liburnia circumcincta Van Duzee, 1909: 203-204.
Megamelus vanduzeei Crawford, 1914: 607, 622.
Megamelus circumcinctus (Van Duzee, 1909); comb. by Crawford 1914: 629.
Liburnia vanduzeei (Crawford, 1914); comb. by Van Duzee 1916: 84.
Liburnia circumcincta Van Duzee, 1909; syn. by Van Duzee 1917: 777.
Delphacodes detecta (Van Duzee, 1897); comb. by Muir and Giffard 1924: 26.
Megamelus vanduzeei Crawford, 1914; syn. by Muir and Giffard 1924: 26.
Delphacodes vanduzeei (Crawford, 1914); comb. by Osborn 1938: 338; Moore 1950a: 257; 1950b: 32.

##### Type locality.

New York City, NY.

##### Diagnosis.

Slightly smaller than *Spartidelphax penedetectus*, with wider vertex (l:w ratio averaging between 1.25–1.31). Aedeagus with 2–3 rows of lateral teeth in distal third on both sides of aedeagus; base of aedeagus abruptly narrowed at about 2/3 length; distal portion of base with fine flange on right side. Parameres in widest view more rounded on outer angle than *Spartidelphax penedetectus*.

*Dimensions.* Male brachypter: body length 2.28 mm (1.89–2.43, n=4), vertex l:w ratio (1.25, n=3), male macropter: body length 3.29 mm (including wings, 2.88–3.67, n=5), vertex l:w ratio (1.33, n=5). Female brachypter: body length 2.89 mm (2.58–3.12, n=4), vertex l:w ratio (1.25, n=3); female macropter: body length 3.61 mm (3.29–4.24, n=5 [paralectotype = 4.24 mm]), vertex l:w (1.31, n=5). Number of calcar teeth 22 (18-24, n=10).

##### Reported hosts.

*Spartina patens* (Aiton) Muhl. (Poaceae, saltmeadow cordgrass), *Spartina alterniflora* Loisel. (smooth cordgrass) (Denno 1977, 1978), with *Spartina alterniflora* “…an inferior host plant for development” (Denno 1977: 366). *Distichlis spicata* (L.) Greene (saltgrass, Poaceae) was reported on specimen labels.

##### Distribution.

USA: CT, DE, FL, GA, LA, MA, MD, ME, MS, NC, NJ, NY, RI, SC, TX, VA, VT; CAN: NS, PE, QC; Anguilla, Bahamas (Exuma, Berry, Eleuthera); Bermuda, British Virgin Islands (Guana, St. Thomas), Jamaica, Mexico, Puerto Rico (inc. Vieques Is.), Turks & Caicos (Bartlett et al. 2014).

##### Remarks.

*Liburnia detecta* Van Duzee, 1897, was described from 2 specimens (1 male, 1 female) from New York City (Van Duzee 1897). The male was designated lectotype by Oman (1947), and at the time both specimens were located in the collection at Iowa State (ISUC). Primary types were subsequently transferred to the National Museum of Natural History (USNM). The lectotype could not be located at either ISUI or USNM, but the female paralectotype was at ISUI.

Beamer (1950: 70) described *Spartidelphax penedetectus* as having “...crown about one-third longer than basal width instead of as wide as long and distinctly narrowed toward apex. Length ♂2.5 mm, ♀3 mm” (for brachypters). Beamer (1950) redescribed *Spartidelphax detectus* did not report body lengths except by quoting Van Duzee (1897: 248), who specified male 3½ mm, female 4 mm for the macropterous syntypes (yielding a length comparison between brachypterous *penedetectus* and macropterous *detectus*). Here we clarify that *penedetectus* is the larger species (*detectus* brachypterous males 2.28 mm, macropterous males 3.29 mm, vs. *penedetectus* brachypterous males 2.33 mm, macropterous males 3.78), although body length does broadly overlap between species. The vertex l:w ratio is approximately 1.25–1.31 for *detectus* and 1.34–1.48 for *penedetectus*. For *penedetectus* Beamer (1950) also noted that crown is narrowed toward the apex. This feature seems valid for the paratypes from Cedar Keys (vertex width near base 0.25, at apex 0.16 versus average measurements of 0.23 near base and 0.22 near apex for *detectus*), but not for other specimens examined.

The most definitive feature that distinguishes the two species is the aedeagus (Fig. 4B, D). In *Spartidelphax detectus* the aedeagus has rows of small teeth on both sides of the apical third, tracing the curve of the aedeagus, with one row extending nearly to the expanded basal portion of the aedeagus. In *Spartidelphax penedetectus*, the aedeagus bears a pair of rows of ventral aedeagal teeth, reduced to fine serrulations in the type series.

Raupp and Denno (1979) found that the density of *Spartidelphax detectus* on *Spartina patens* exceeded 400 per kg of live grass sampled over a 6-month period, and was described as a dominant herbivore on *Spartina patens* by Denno (1977). It appears to have 3 non-synchronous generations per year in New Jersey, and overwinters as 4^th^ or 5^th^ instar (Denno 1976, 1977). Populations are wing polymorphic (both brachypters and macropters present within a population), with proportions of wing brachyptery and macroptery varying based on complex interactions of seasonal, environmental and population variables. An overall annual brachyptery rate of 86% (out of 23,868 specimens) was reported by Denno (1980) in New Jersey. Denno (1980) described niche differentiation among sap-feeding taxa on *Spartina patens*, including *Spartidelphax detectus*.

##### Type material examined.

Paralectotype. *Liburnia detecta* Van Duzee, 1897 (female, ISUC) “[blank ‘purple’ tab] // E.B. Southwick // ♀ // type // Liburnia / detecta Van D. [handwritten] // UDCC_TCN 00017671 [2D barcode]” (reported by Van Duzee 1897 as from New York City).

##### Other material examined.

**USA: Connecticut: *New London Co.*:** Mystic, 19 Aug 1934, P. W. Oman (1f, 1m; USNM). **Delaware: *Kent Co.*:** Dover, 25 Aug 1927, H. L. Dozier (1m; UDCC); Little Creek, Port Mahon Road, 19 Aug 1999, C. R. Bartlett (1m; UDCC); Pickering Beach, 19 Aug 1999, C. R. Bartlett (1m, 12f; UDCC); Taylors Bridge, Jul 1999, C. R. Bartlett (10f, 4m; UDCC); near Fleming’s Landing, Rt. 9 near Leipsic River, C. R. Bartlett (5f; UDCC); near Port Mahon, 19 Aug 1999, C. R. Bartlett (1m; UDCC); near Woodland Beach, 07 Jul 1999, R. L. Snyder (4m, 9f; UDCC); ***New Castle Co.*:** Middletown, Brick Mill Farm; 522 St Michael Drive, 28 Aug 2003, A. Gonzon (1m; UDCC); Newark, UD farm, Wildlife Refuge, 18 May 2009, C. R. Bartlett (1m; UDCC). near Woodland Beach, 07 Aug 1994, C. R. Bartlett (15m, 13f; UDCC); ***Sussex Co.*:** Bayard, Assawoman Wildlife Management Area, 11 Sep 2010, M. A. Johnston (1m; UDCC); Rehoboth Beach, 30 Aug 1921, H. G. Dyar (2m; USNM); South Bethany, Assawoman Wildlife Area, 29 Jun 2002, C. R. Bartlett (1f, 1m; UDCC); Thompson’s Island, 0.25mi from trailhead, 09 Sep 2004, A. Gonzon (1m, 1f; UDCC); near Lewes, Oyster Rocks Road, 06 Jul 1994, C. R. Bartlett (8m, 5f; UDCC). **Florida: *Duval Co.*:** Paradise Key, Jacksonville, 10 Apr 1921, D. M. DeLong (2m; UDCC); ***Franklin Co.*:** Bald Point, near Panacea, 27 Jul 2000, C. R. Bartlett (2f, 12m; UDCC); ***Hillsborough Co.*:** Tampa, 01 Nov 1928, E. D. Ball (1m; USNM); ***Miami-Dade Co.*:** Miami Beach, Apr 1937 (1m, 1f; NCSU); ***Seminole Co.*:** Sanford, 1 m, 29 Oct 1926, E. D. Ball (1m; USNM). **Louisiana: *Cameron Parish*:** Cameron, 1 m, 20 Jun 1930 (3m, 2f; NCSU). **Maryland: *Anne Arundel Co.*:** 6 km S Edgewater SERO, 15 Jun 1976, J. H. Falk (1m; USNM); ***St. Mary’s Co.*:** 2.3 mi E of Piney Point, 1 m, 12 Jul 1931, P. W. Oman, *Spartina patens* (1m, 1f; USNM); Piney Point, 26 Aug 1946, R. I. Sailer (1m; USNM). **Massachusetts: *Barnstable Co.*:** Falmouth, 17 Jul 1926 (1f, 2m; USNM); Woods Hole, 3 m, 10 Jul 1925, E. D. Ball (1m; USNM). **Mississippi: *Jackson Co.*:** Pascagoula, 30.3484°N, 88.55655°W, 3 m, 08 Aug 1921 (1m; ISUI). **New Hampshire: *Rockingham Co.*:** Rye Beach, 11 Aug 1985, G. F. and J. F. Hevel (2m; USNM); Rockingham, Odiorne Point State Park, 43.04791, -70.71871; 13 Aug 2008, D. S. Chandler (2m, 3f; DENH). **New Jersey: *Gloucester Co.*:** Williamstown, 43 m, 14 Sep 2009, A. M. Colavecchio (1f; UDCC); ***Salem Co.*:** 166 Maskells Mill Road, 16 Aug 2000, C. R. Bartlett & F. Robbins (5f; UDCC). **North Carolina: *Brunswick Co.*:** Bald Head Island, Bald Head Creek, 02 Jul 2007, N. H. Nazdrowicz (1m, 2f UDCC); Southport, 28 Jul 1919, Osborn & Metcalf (1m, 3f; NCSU); 10 Oct 1948, C.W. Sabrosky (1m; USNM); ***Carteret Co.*:** near Atlantic, 29 Sep 1973, N. Newton (6f, 5m; UDCC); ***Dare Co.*:** Bodie Island, 14 Jun 1989, R. L. Blinn (3f; NCSU); ***Hyde Co.*:** Ocracoke Island, 2 m, 25 Aug 1962, T. Daggy (1m; NCSU); 15 Jun 1976, N. Newton (1m; UDCC); ***New Hanover Co.*:** Carolina Beach, May 1934, Z. P. Metcalf (19f, 29m; NCSU); Fort Fisher, 28 Oct 1934, Z. P. Metcalf (2m; NCSU); Wrightsville Beach, 27 Jul 1919, Osborn & Metcalf (21f, 11m; NCSU); ***Onslow Co.*:** Ashe Island, 04 Jun 1975, J. C. Dukes, *Distichlis spicata* (26m, 13f; NCSU); 19 Aug 1975, J. C. Dukes, *Spartina patens* (2m; NCSU); 15 Jun 1976, T. D. Edwards (1m; NCSU); 21 Jun 1976, T. D. Edwards (1f, 1m; NCSU); ***Pender Co.*:** Burgaw, May 1925, [Spartina] patens (1m; NCSU). **South Carolina: *Charleston Co.*:** Charleston, 02 Jul 1958, D. A. Young (2m; NCSU); 10 Jul 1958, D. A. Young (1m NCSU). **Texas: *Cameron Co.*:** Brownsville, 11 Mar 1936, P. A. Glick (1m; USNM). **Virginia: *Hampton Co.*:** Hampton, Jul 1908 (1m, 3f; URIC); ***Northampton Co.*:** Cape Charles, 31 Jul 1920, D. M. DeLong (3f, 1m; NCSU); ***Virginia Beach Co.*:** Cape Henry, 03 Jul 1938, P. W. Oman (2m; USNM). **PUERTO RICO:** Vieques Island, 23 Oct 1947, J. S. Caldwell, 1f (USNM). **VIRGIN ISLANDS (BRITISH): Guana Island:** North Beach, 18.48178°N, 64.57515°W, 25 Oct 2012, A. G. Wheeler (2m, 2f; UDCC). **BAHAMAS: Exuma Cays**, Leaf cays of Allen cays, 07 Jan 1953, E. B. Hayden, Van Voast AMNH Bahama Islds. Exped. (12m, 4f, AMNH); **Eleuthera Island**, New Portsmouth (Rock Sound District), 28 Mar 1953, E. B. Hayden & L. Giovannoli, Van Voast AMNH Bahama Islds. Exped. (1m, AMNH).

#### Spartidelphax luteivittus

Taxon classificationAnimaliaHemipteraDelphacidae

(Walker, 1851) comb. n.

Figures 6 , 7

Delphax luteivitta Walker, 1851: 354.
Dicranotropis (?) luteivitta (Walker, 1851); comb. by Van Duzee 1916: 84.
Stenocranus luteivitta (Walker, 1851); comb. by Muir and Giffard 1924: 12; to *incertae sedis* by Beamer (1946: 1).
Delphacodes luteivitta (Walker, 1851); comb. by Bartlett 2010: 472.

##### Type locality.

Florida, Duval County, St. Johns Bluff.

##### Remarks.

**Figure 6.**
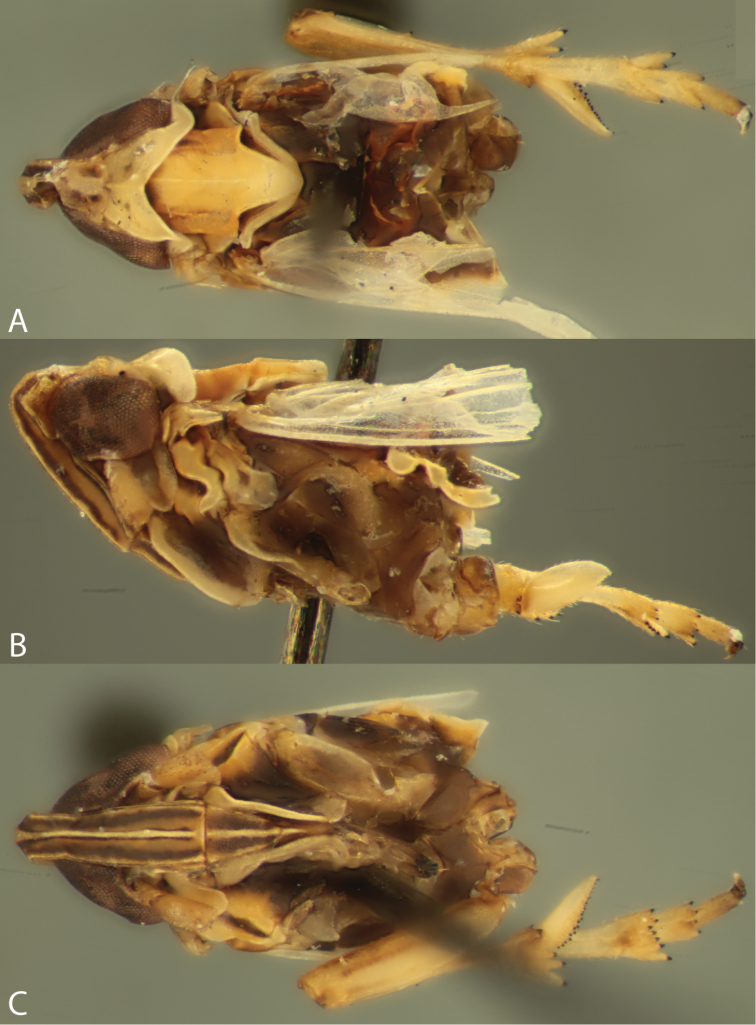
Holotype of *Delphax luteivitta* Walker, 1851. **A** dorsal view **B** left lateral view **C** ventral view.

**Figure 7.**
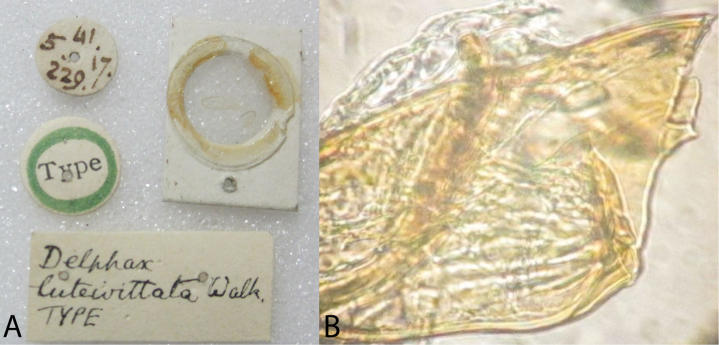
Labels and aedeagus of *Delphax luteivitta* Walker, 1851 (holotype). **A** specimen labels and forewing card mount **B** base of aedeagus (obscured by unidentified adhered membrane).

The male holotype of *Delphax luteivitta* (at BMNH) is in poor condition (Figs 6–7). The specimen is shriveled and damaged, making the proportions of the head suspect. The coloration and habitus are similar to the other species of *Spartidelphax*. The wings are frayed and fragmentary with the forewing of only one side complete (mounted on specimen card, Fig. 7A). The abdomen has been removed for dissection, and only portions of the abdomen remain. The aedeagus (Fig. 7) although similar to the other species of *Spartidelphax* is missing the distal third, which bears the most definitive features separating *Spartidelphax detectus* and *Spartidelphax penedetectus*, with much of the base obscured by an adhered membrane.

##### Type material examined.

Holotype *Delphax luteivitta* Walker, 1851 (male, BPBM) “5 41 17 229 (circular label, reading clockwise, meaning entry 229 of May 17, 1841)) // Type (circular label, green boarder) // Delphax / luteivittata [sic] Walk. / TYPE (handwritten)”.

## Discussion

*Spartidelphax detectus* and *Spartidelphax penedetectus* are closely allied species. The lack of published records of *Spartidelphax penedetectus* on the Atlantic coast may be because of the great similarity of these species, the numerical over-dominance of *Spartidelphax detectus* in coastal marshes, and that most records of *Spartidelphax penedetectus* were from the Gulf coast, so planthopper workers may not have expected, or sought, *Spartidelphax penedetectus* on the Atlantic coast. Targeted collections on *Spartina alternifolia* should find *Spartidelphax penedetectus* throughout the Atlantic coast.

Our original intention was to determine whether *Spartidelphax luteivittus* was a senior synonym or a valid species. The very poor condition of the holotype obscured all of the most useful features distinguishing *Spartidelphax detectus* from *Spartidelphax penedetectus*, and also did not obviate the possibility that *Spartidelphax luteivittus* represents a third valid *Spartidelphax* taxon. We also studied morphological variation within the species over the geographic distribution of *Spartidelphax*, and found variation in size, shape details of the parameres, armature of the diaphragm, and shape and serration of the aedeagus; but were able to attribute males of all the examined specimens to either *Spartidelphax detectus* or *Spartidelphax penedetectus*. However, a field investigation to collect *Spartidelphax* from the different species of *Spartina* (including species not yet implicated as hosts such as *Spartina bakeri* Merr., *Spartina cynosuroides* (L.) Roth, *Spartina pectinata* Bosc ex Link, and *Spartina spartinae* (Trin.) Merr. ex Hitchc.) is needed to determine if there are additional species of *Spartidelphax*. In the meantime *Spartidelphax luteivittus* is best treated as a *nomen dubium*.

## Supplementary Material

XML Treatment for Spartidelphax

XML Treatment for Spartidelphax penedetectus

XML Treatment for Spartidelphax detectus

XML Treatment for Spartidelphax luteivittus
